# Supplementary material for: The Patterns and Appropriateness of Systemic Antifungal Prescriptions in a Regional Hospital in Hong Kong
Source: Antibiotics (Basel). 2025 May 29;14(6):556. doi: 10.3390/antibiotics14060556 (PMC12189983; doi:10.3390/antibiotics14060556)
Supplement: Supplementary file 1 [file antibiotics-14-00556-s001.zip › antibiotics-3643814 Supplementary Materials-main.pdf]

Supplementary Materials

Supplementary Table S1. Definition of antifungal therapeutic strategies in this study

Prophylactic	Antifungal treatment initiated in patients at high risk of IFD but without any clinical, radiological or mycological evidence of IFD
Empirical	Antifungal treatment initiated over clinical suspicion of fungal infection but there was neither radiological nor mycological evidence of IFD
Pre-emptive	Early antifungal treatment in patients with risk factors of IFD, clinical and/or radiological signs suggestive of IFD but lacked mycological evidence of IFD (e.g., possible IFD per EORTC/MSG criteria)
Targeted	Antifungal treatment in patients with risk factors of IFD, clinical and/or radiological signs suggestive of IFD and supportive mycological and/or histological evidence of IFD (i.e., proven or probable IFD per EORTC/MSG criteria)<br>Clinical diagnosis of superficial candidiasis and dermatophytosis

Supplementary Table S2. Criteria used to assess adherence to antifungal treatment guidelines in this study

Assessment	Indication	Dosage	Duration	Antifungal-concomitant drug interaction
Appropriate	Follow SPC and/or published guidelines, and adapted to mycological data	Appropriate dose within 10% under- or overdose margin, AND Loading dose given when recommended	Within 10% margin of treatment duration suggested by published guidelines and available literatures	Antifungal has no potential interaction with concomitant medications, or Antifungal has potential interactions with concomitant medications of mild to moderate consequences and is subjected to clinical monitoring and/or dose adjustment if required
Debatable	Choice of antifungal not recommended by SPC and/or guidelines but there is no available alternative	Under- or overdose by 10-25%, and/or Absence of loading dose, and/or Dosage form not recommended by guidelines	Inadequate or excessive duration by 10-25% compared to suggestions from published guidelines and available literatures	Antifungal has potential interactions with concomitant medication of mild to moderate consequences but there is no clinical monitoring and/or dose adjustment when required
Inappropriate	Inappropriate choice based on SPC, guidelines or mycological results with the existence of an available alternative	Under- or overdose by > 25%, and/or No discontinuation or dose adjustment in case of clinically related adverse events, and/or No TDM when locally available	Inadequate or excessive duration by > 25% compared to suggestions from published guidelines and available literatures, and/or Fail to take off empirical antifungal when alternative diagnosis was made	Antifungal has potential interactions with concomitant medications of potentially serious consequences, including adverse drug effects and treatment failure but there is no clinical monitoring and therapy modifications, and/or Concomitant use of antifungals of the same class

Supplementary Table S3. Antifungal prescriptions with a positive mycological culture N = 55[†]

	N	%
**Yeast**	**51**	**92.7**
Candida albicans	19	34.5
Candida glabrata	9	16.4
Candida parapsilosis	8	14.5
Candida tropicalis	5	9.1
Candida krusei	2	3.6
Candida lusitaniae	1	1.8
Kodamaea ohmeri	5	9.1
Trichosporon faecale	2	3.6
**Mold**	**8**	**14.5**
Aspergillus fumigatus	5	9.1
Penicillium spp.	2	3.6
Mucor spp.	1	1.8
**Dimorphjc fungi**	**2**	**3.6**
Talaromyces marneffei	2	3.6
**Total**	**61**[a]	

†A patient may have more than one positive mycological culture leading up to antifungal prescription.

Supplementary Table S4. Distribution of antifungal prescriptions across departments/specialties in PYNEH from May 1 to July 31, 2023

		**Antifungal drug N (% of specialty total)**										
**Department/ Specialty**	**Prescriptions N (%)**	Fluconazole	Itraconazole	Voriconazole	Isavuconazole	Posaconazole	Amphotericin B Deoxycholate	Liposomal Amphotericin B	Micafungin	Anidulafungin	Terbinafine	Flucytosine
Haematology	84 (47.5%)	5 (6.0%)	54 (64.3%)	5 (6.0%)	-	3 (3.6%)	-	-	17 (20.2%)	-	-	-
Infectious Diseases	22 (12.4%)	6 (27.2%)	1 (4.5%)	4 (18.2%)	-	-	-	-	9 (40.9%)	2 (9.1%)	-	-
Nephrology	5 (2.8%)	2 (40%)	-	1 (20%)	-	-	1 (20%)	-	-	-	-	1 (20%)
Respiratory medicine	1 (0.6%)	-	1 (100%)	-	-	-	-	-	-	-	-	-
Medicine (Other)	26 (14.7%)	6 (23.1%)	-	3 (11.5%)	1 (3.8%)	-	-	2 (7.7%)	9 (34.6%)	-	5 (19.2%)	-
Intensive Care Unit	16 (9.0%)	3 (18.8%)	-	1 (6.3%)	-	-	-	-	11 (68.5%)	1 (6.3%)	-	-
Cardiac Care Unit	1 (0.6%)	1 (100%)	-	-	-	-	-	-	-	-	-	-
Neonatal Intensive Care Unit	5 (2.8%)	5 (100%)	-	-	-	-	-	-	-	-	-	-

Clinical oncology	4 (2.3%)	2 (50%)	-	-	-	-	-	-	2 (50%)	-	-	-
Dermatology	2 (1.1%)	-	-	-	-	-	-	-	-	-	2 (100%)	-
Neurosurgery	1 (0.6%)	1 (100%)	-	-	-	-	-	-	-	-	-	-
Orthopaedics	1 (0.6%)	-	-	-	-	-	-	1 (100%)	-	-	-	-
Otorhinolaryngology	1 (0.6%)	-	-	-	-	-	-	-	1 (100%)	-	-	-
Surgery	8 (4.5%)	2 (25%)	-	-	-	-	-	1 (12.5%)	4 (50%)	1 (12.5%)	-	-
Total	177 (100%)	33	56	14	1	3	1	4	53	4	7	1

Supplementary Table S5. Antifungals prescriptions with inappropriate indications (N = 48)

**Reason**	**N**	**Antifungal**
Mycological results represent colonization or contamination	18	Micafungin (9), Fluconazole (4), Anidulafungin (2), Voriconazole (2), Itraconazole (1)
No evidence to suggest IFD, IPA or CAPA	15	Micafungin (10), Fluconazole (2), Voriconazole (2), Terbinafine (1)
Inappropriate antifungal choice	11	Micafungin (7), Fluconazole (2), Voriconazole (1), Terbinafine (1)
Redundant antifungals	4	Fluconazole (1), Itraconazole (1), Voriconazole (1), Flucytosine (1)

Supplementary Table S6. Antifungal prescriptions with debatable or inappropriate dosage (N = 76)

**Debatable antifungal dosage (N= 54)**		
**Reason**	**N**	Antifungal
Oral IFD prophylaxis with daily itraconazole 200mg capsules	52	Itraconazole (52)
No loading dose	2	Fluconazole (2)
**Inappropriate dosage (N = 22)**		
Reason	N	Antifungal
No therapeutic drug monitoring	8	Voriconazole (8)
Underdose	7	Fluconazole (4), Voriconazole (1), Flucytosine (1), Terbinafine (1)
Continue systemic antifungal without modifying therapy despite adverse drug effect	5	Fluconazole (3), Itraconazole (2)
Overdose	2	Fluconazole (1), Terbinafine (1)

Supplementary Table S7. Antifungal prescriptions with inappropriate duration (N = 15)

**Reason**	**N**	**Antifungal**
Excessive duration	12	Micafungin (4), Fluconazole (3), Voriconazole (2), Itraconazole (1),

		Isavuconazole (1), Liposomal Amphotericin B (1)
Inadequate duration	3	Fluconazole (2), Terbinafine (1)

Supplementary Table S8. Significant antifungal-concomitant drug interactions in this study (N = 169)

Antifungal	Risk rating†	Interacting drug (frequency)
Fluconazole (N =52)	D	Alprazolam (4), Rifampicin (2), Amiodarone (1), Clopidogrel (1)
	C	Lansoprazole (8), Amlodipine (6), Atorvastatin (6), Dexamethasone (5), Levofloxacin (4), Apixaban (2), Dasatinib (2), Gliclazide (2), Losartan (2), Alfuzosin (1), Amitriptyline (1), Ciprofloxacin (1), Olanzapine (1), Pantoprazole (1), Simvastatin (1), Tramadol (1)
Itraconazole (N = 77)	X	Aprepitant (3), Phenytoin (2)
	D	Pantoprazole (20), Calcium carbonate (12), Atorvastatin (5), Venetoclax‡ (5), Dasatinib (3), Digoxin (1), Lansoprazole (1)
	C	Amlodipine (4), Dexamethasone (4), Mirtazapine (3), Gliclazide (2), Tramadol (2), Venlafaxine (2), Clofazimine (1), Clonazepam (1), Etoposide (1), Levofloxacin (1), Morphine (1), Oxybutynin (1), Prednisolone (1), Sertraline (1)
Voriconazole (N = 28)	X	Amiodarone (1), Pazopanib (1), Rifampicin (1)
	D	Ibrutinib (1), Venetoclax (1)
	C	Pantoprazole (5), Dexamethasone (3), Amlodipine (2), Clopidogrel (2), Ethambutol (2), Lansoprazole (2), Levofloxacin (2), Clindamycin (1), Hydrocortisone (1), Melatonin (1), Tramadol (1), Zolpidem (1)
Isavuconazole (N = 1)	C	Dexamethasone (1)
Posaconazole N = 2	D	Pantoprazole (2), Lansoprazole (1)
	C	Diltiazem (1)
Amphotericin B Deoxycholate (N = 1)	C	Vancomycin (1)
Liposomal Amphotericin B (N = 4)	C	Amlodipine (1), Frusemide (1), Losartan (1), Valganciclovir (1)
Terbinafine (N = 2)	C	Tramadol (2)

† Risk rating C: monitor therapy, D: consider therapy modification, X: Avoid combination.

‡ Itraconazole prescribed by haematology team designated specifically to potentiate Venetoclax is considered to be appropriate.

Supplementary Table S9. Appropriateness of systemic antifungal prescription across different specialties.

**Specialties**	**Appropriate (%)**	**Inappropriate (%)**	**Debatable (%)**	**Subtotal**
Haematology	15 (17.9%)	48 (57.1%)	21 (25%)	84
Infectious disease	2 (9.1%)	20 (90.9%)	-	22
Nephrology	2 (40%)	3 (60%)	-	5
Respiratory medicine	-	1 (100%)	-	1
Medicine (Other)	10 (38.5%)	16 (61.5%)		26
Intensive Care Unit	4 (25%)	12 (75%)	-	16
Cardiac Care Unit	-	1 (100%)	-	1
Neonatal Intensive Care Unit	5 (100%)	-	-	5
Clinical oncology	2 (50%)	2 (50%)	-	4
Dermatology	2 (100%)	-	-	2

Neurosurgery	-	1 (100%)	-	2
Orthopaedics	1 (100%)	-	-	1
Otorhinolaryngology	1 (100%)	-	-	1
Surgery	5 (62.5%)	3 (37.5%)	-	8
**Total**	**49 (27.7%)**	**107 (60.5%)**	**21 (11.9%)**	**177**
